# Rabbit as an animal model for the study of biological grafts in pelvic floor dysfunctions

**DOI:** 10.1038/s41598-021-89698-z

**Published:** 2021-05-18

**Authors:** Marta Peró, Laura Casani, Cristina Castells-Sala, Maria Luisa Pérez, Esther Moga Naranjo, Oriol Juan-Babot, Leticia Alserawan De Lamo, Patricia López-Chicón, Anna Vilarrodona Serrat, Lina Badimon, Oriol Porta Roda

**Affiliations:** 1 Department of Obstetrics and Gynecology, Hospital de la Santa Creu i Sant Pau, Universitat Autònoma de Barcelona, Carrer Sant Quintí, 89, 08041 Barcelona, Spain; 2 Research Institute of the Hospital de Sant Pau-IIB Sant Pau, Barcelona, Spain; 3 Barcelona Tissue Bank, Banc de Sang i Teixits (BST), Barcelona, Spain; 4 Biomedical Research Institute (IIB-Sant Pau; SGR1113), Barcelona, Spain; 5 Department of Immunology, Hospital de la Santa Creu i Sant Pau, Biomedical Research Institute Sant Pau (IIB Sant Pau), Universitat Autònoma de Barcelona, Barcelona, Spain

**Keywords:** Immunohistochemistry, Animal disease models, Urogenital reproductive disorders, Experimental models of disease, Preclinical research, Translational research, Biomedical materials, Implants, Tissues, Urinary incontinence

## Abstract

The aims of this study were to evaluate the feasibility of the New Zealand White (NZW) rabbit for studying implanted biomaterials in pelvic reconstructive surgery; and to compare the occurrence of graft-related complications of a commercial polypropylene (PP) mesh and new developed human dermal matrix implanted at vaginal and abdominal level. 20 white female NZW rabbits were randomized into two groups, experimental group (human acellular dermal matrices-hADM-graft) and control group (commercial PP graft). In each animal, grafts were surgically implanted subcutaneously in the abdominal wall and in the vaginal submucosa layer for 180 days. The graft segments were then removed and the surgical and clinical results were analyzed. The main surgical challenges during graft implantation were: (a) an adequate vaginal exposure while maintaining the integrity of the vaginal mucosa layer; (b) to keep aseptic conditions; (c) to locate and dissect the breast vein abdominal surgery; and (d) to withdraw blood samples from the ear artery. The most abnormal findings during the explant surgery were found in the PP group (33% of vaginal mesh extrusion) in comparison with the hADM group (0% of vaginal graft extrusion), *p* = 0.015. Interestingly, macroscopic observation showed that the integration of the vaginal grafts was more common in the hADM group (40%) than in the PP group, in which the vaginal mesh was identified in 100% of the animals (*p* = 0.014). The NZW rabbit is a good model for assessing materials to be used as grafts for pelvic reconstructive surgery and vaginal surgery. Animals are easily managed during the procedures, including surgical intervention and vaginal mucosa approach. Additionally, hADM is associated with fewer clinical complications, as well as better macroscopic tissue integration, compared to PP mesh.

## Introduction

Pelvic floor dysfunctions (PFD) such as pelvic organ prolapse (POP), or urinary incontinence (UI) are common conditions that affect a third of the adult female population^1^. In these frequent dysfunctions, surgical treatment has shown good results. The surgical repair may involve the use of non-absorbable, synthetic prostheses, usually polypropylene (PP). PP meshes have been associated with severe complications such as erosion, retraction, and pain^2^.

In July 2018, the use of mesh implants to treat stress urinary incontinence was suspended by the National Health Service (NHS) of the United Kingdom^3^. And in April 2019 the United States (US) Food and Drug Administration (FDA) banned vaginally-placed mesh implants for treating pelvic organ prolapse^4^. With this growing concern for safety, there is a worldwide agreement on the need for research and innovation to find alternative materials to be used in pelvic reconstructive surgery.

This need of new materials with an effective and safe mesh design has approached the development of acellular matrices (AM). AMs represent a new generation of biocompatible materials processed to obtain a decellularized scaffold of fibers whose architecture and extracellular matrix remain intact^5–7^.

In recent years, many different types of biological meshes have been marketed and their efficacy evaluated^8,9^. Specifically, human acellular dermal matrices (hADM), available in the US for more than 15 years, have been used in more than 2 million implant procedures and information is available on its clinical safety and efficacy in different clinical applications^10,11^. Reconstructive surgeries such as chronic wounds closure, immediate breast reconstruction, abdominal wall and hernia repair, and tendon reinforcement are likely to use dermal matrices^10,12,13^.

However, the gynecological application of dermal matrices has been poorly evaluated^14^, and there is limited information about the behaviour of vaginally applied hADM. Therefore, it is required to carry out preclinical studies to assess hADM in the repair of PFD. In this way, international associations such as the *International Urogynecological Association* (IUGA) have presented a consensus document that specify the steps to follow for the introduction of new devices to be used in prolapse surgery, and they recommend to previously perform preclinical studies in animals for the evaluation of the host's inflammatory response^15^.

The election of the most suitable animal model to obtain clinical results after placing the hADM at the vaginal level requires to fulfill different objectives and conditions. The most studied animals are mice, rat, rabbit, sheep, pig, and non-human primates (NHP). Published studies show that several models can be used, and there is no animal that is perfect for this purpose. Each one has its own benefits and weakness^16–18^.

We selected the New Zealand White (NZW) rabbit as animal model because of its adequate life expectancy for the duration of the study, it has perineal musculature associated with the urogenital tract similar to humans^19,20^, and has appropriate vaginal size to perform a vaginal graft placement. Additionally, the rabbits are economical and easily housed, handled and anaesthetized.

Another advantage of this model is that it allows the study of implants in the vaginal and abdominal location in the same animal concomitantly, with the aim of evaluating whether clinical changes appear depending on the implant location.

Other animals have a reproductive and urinary pathophysiology more similar to humans than rabbits, such as ewes or NHP^16–18^. These models would probably be better for studying the physiology of POP or UI. However, this research does not pretend to study the therapeutic efficacy of hADM. Instead, it is dedicated to demonstrating the occurrence of local complications, such as exposure or infection, especially at vaginal location. Additionally, the scientific literature shows many examples of experimental studies using NZW rabbits for the evaluation of new biomaterials for the treatment of PFD^21–31^.

This study aims to describe the surgical complexity of the NZW rabbit model, their clinical monitoring, as well as the standardization of the model. It includes the description of the surgical difficulties of implanting prostheses at the vaginal and abdominal level in rabbits, as well as the difficulties in housing them, and the occurrence of graft-related complications in different locations.

This information could guide future works designed to test devices for vaginal application and will help other groups that focus their research in the urogynecology area.

## Material and methods

### Experimental design and subjects of study

The study was performed by Barcelona Tissue Bank (BTB), the Hospital de la Santa Creu i Sant Pau, and at the Research Institute of the Hospital de Sant Pau-IIB Sant Pau.

The study protocol was approved by the Internal Animal Care and Use Committee (CEEA-IRHSCSP) and the competent government authority (Generalitat de Catalunya, Animal Experimentation Commission, project number 9669). All animal procedures were carried out in strict accordance with the guidelines of Directive 2010/63/EU of the European Parliament on the protection of animals used for scientific purposes. In addition, we followed the ARRIVE guidelines and committed ourselves to the 3Rs of laboratory animal research. The animal experimental project was performed in the Animal Experimental Service of the accredited IRHSCSP, ISO 9001:2015 accredited.

This study followed the ethical precepts of the Declaration of Helsinki and was approved by local ethics committee. Human tissue was processed according to guidance for clinical use (EEC regulations 2004/23/CE and 2006/17/CE) and to the legal requirements for the use of biological samples for research in Spain (Law 14/2007 and RD 1716/2011). Ethics institutional review board (IRB) approval was obtained (CEIm Hospital Valle Hebrón, Barcelona; PR (BST)314/2019). In all cases, informed consent was obtained from the donors' relatives.

A total of 20 female multiparous NZW rabbits were randomly allocated to receive control (PP mesh) or experimental (hADM) grafts.

Each rabbit received 4 grafts: 2 grafts in the vaginal submucosa layer and two in the subcutaneous tissue of the abdominal wall, over the muscular fascia.

Regarding the vaginal grafts, one (5 × 5 mm) was placed in the anterior vaginal wall and used for histological and immunohistochemical studies. The other one (10 × 5 mm) was placed in the submucosa of the posterior vaginal wall and was used to perform the biomechanical study.

The size of the abdominal grafts was the same as that of the vaginal grafts, but both were stitched together in the right caudal quarter of the abdominal wall.

The implants were removed 180 days later, at which time the animals were also humanely euthanized.

### Graft preparation

#### Preparation of hADM samples

hADM was obtained from skin tissue procured from the back and lower limbs of a human cadaveric donor by manual dermatome. The tissue was processed in clean rooms in accordance with Good Manufacturing Practices (GMP) regulations in the BTB. The processing consisted of, first, the selection of the homogeneous fragments in thickness and their decontamination in antibiotic solution for 16–24 h, and then their decellularization. Decellularization was achieved by chemical, biological and mechanical treatment as follows: the skin was incubated in hypertonic solution, which led to the cellular lysis, then an incubation in a proteolytic enzyme, resulting in the removal of the genetic material, and a final incubation in anionic surfactant for the washing out of the cellular debris. In order to remove any reagent, 10 washes were carried out in 0.9% NaCl. The 10 × 5 mm samples were prepared and stored in glycerol solution in a double bag at room temperature until use. To ensure strict microbiological control, several microbiological controls were performed throughout the graft processing.

#### PP graft preparation

The material (Gyneband, Mallanets, Spain) was delivered in a commercial sterile container, ready for medical use in humans. Under conditions of surgical asepsis, it was removed from the container and cut into 10 × 5 mm and 5 × 5 mm pieces immediately before proceeding to the implant surgery.

### Surgical procedure

Animals were anesthetized with ketamine (15 mg/kg subcutaneous; sc) and medetomidine (0.5 mg/kg sc). Each rabbit received a prophylactic antibiotic dose (Ceftiofur 50 mg/kg sc) and nonsteroidal anti-inflammatory drug (meloxicam 1 mg/kg intramuscular -im-).

Before starting the surgery, the areas of surgical incision were shaved and disinfected, and blood samples (6 cc) were obtained from the ear artery to study inflammatory markers. Serial blood samples were obtained on days: 0 (day of implantation surgery, 7, 30 and 180 (day of euthanasia).

#### Abdominal implants

**Figure 1:**
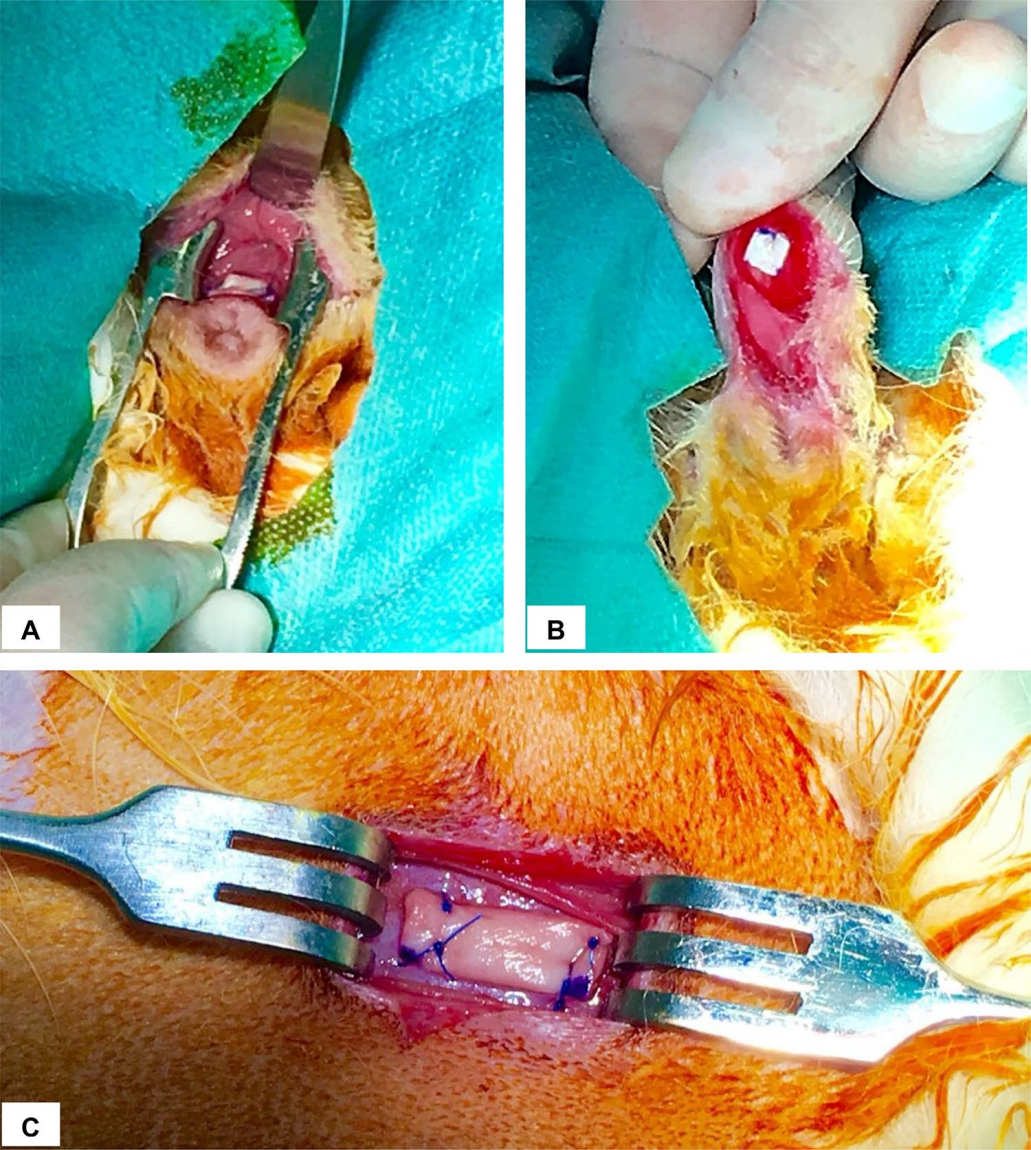
Vaginal and abdominal implant placement in NSW rabbit model. (**A**) hADM fixed in posterior vaginal submucosal layer. (**B**) hADM fixed in anterior vaginal submucosal layer. (**C**) hADM fixed in the subcutaneous tissue of the abdominal wall.

A transverse incision was made in the abdominal midline, at the level of the intermammary line of the last two nipples on the right side of the rabbit, to expose the anterior abdominal fascia. Both fragments of the graft (hADM or PP) were positioned and fixed with prolene (Ethicon) 5/0 discontinuous suture (Fig. 1C). The abdominal wall was closed with 4/0 vicryl rapide (Ethicon) thread in two layers: continuous suture for subcutaneous tissue, and continuous intradermal suture for skin tissue.

#### Vaginal implants

A transverse incision was made in the anterior vaginal wall, approximately 1 cm from the vaginal entrance. The vaginal mucosa layer was dissected and the 5 × 5 mm graft was implanted and fixed with the same procedure as in the abdominal implant (Fig. 1B). The same procedure was repeated on the posterior vaginal wall, using the 10 × 5 mm graft (Fig. 1A).

The vaginal mucosa was closed with a 4/0 vicryl thread using an interrupted suture.

Once the implants had been placed, a preventive dose of buprenorphine (0.01 mg/kg, sc) was administered. To avoid licking and infection of the wound, rabbits wore a protective collar for 7 days after surgery. Animals could move freely in their pens and were under a strict veterinary control. During the entire period of study animals were supervised daily and weighted weekly and complications related to the implant were closely monitored.

After 180 days, rabbits were anesthetized as described before and the grafts were explanted, removing the prosthesis together with surrounding tissue.

Animals were euthanized under deep anesthesia according to the protocol by administration of 150 mg/kg intravenous pentobarbital.

### Variables and parameters investigated at surgery and at follow-up

#### Surgical variables

Surgical time duration, complications and difficulties were collected by the investigator during the implant surgery.

#### Clinical complications during animal follow-up

Signs of pain/stress in the animals were evaluated by the Grimace Scale. This scale allows an objective evaluation of animals' pain and distress through their facial expression, especially orbital closure, the flattening of the cheeks, angulation of the nostrils, stiffness of the whiskers and subsequent rectification of the ears. Each item was scored from 0 to 3, as follows: 0 = not present, 1 = moderately present and 2 = obviously present.

Pain/discomfort (any value > 1) was treated with an additional painkiller dose (buprenorphine, 0.01 mg/kg, sc).

Signs of loss of well-being: anxiety, depression, inactivity, restlessness, shrieks, or groans, grinding of teeth, tonic immobility, rejection of water and/or food, weight loss were also surveyed. Clinical signs of surgical site complication were equally inspected and registered.

#### Macroscopic observation of explants

During explant surgery, the macroscopic aspect of the explants was evaluated with particular interest in the presence of: (a) evidence of seroma (accumulation of serous fluid around the graft); (b) signs of local infection (erythema or purulent suppuration); and, (c) evidence of extrusion of the graft (skin necrosis or dehiscence of the surgical wound with exposure of the graft).

### Statistical analysis

As descriptive data analysis we used the median, and also the mean with standard deviation. The relationship between categorical variables was analyzed using the corresponding contingency tables, calculating the percentage in each group and application of chi-square test with the approximation of the probability ratio. In the ordinal variables, the comparison between two groups was made with non-parametric Mann–Whitney test. In all cases, the usual level of significance was 5% (alpha = 0.05). All analyses were performed with the statistical IBM-SPSS package (V25).

## Results

Twenty animals were included, 10 in the experimental group (hADM), and 10 in the control group (PP). During the study (83 days after surgery), one rabbit in the control group died due to causes not related to the grafts.

### Surgical challenges during surgical graft implantation

#### Exposure of the vaginal surgical field

Due to the small size of the surgical field, a recurring difficulty was the vaginal exposure. This challenge was overcome by placing an eyelid retractor in the vaginal introitus. The rest of the instruments used were the standard microsurgical devices.

#### Integrity of the vaginal mucosa layer

Grafts were implanted at the level of the vaginal submucosa layer, so a meticulous vaginal dissection was needed. Because it is an extremely thin layer, another difficulty in most of the surgeries performed was to maintain the integrity of the vaginal mucosa layer during the graft implantation. The maintenance of this layer is crucial to reduce the risk of future implant extrusions. Despite these difficulties, the preservation of the mucosa was successfully obtained in all animals.

#### Aseptic conditions

It was difficult to keep aseptic conditions due to the large amount of hair in this animal model. The methods used to achieve adequate asepsis in the surgical field were an extensive shaving of the NZW’s abdomen and external genitalia, and a careful and precise handling of the animals during surgeries.

#### Location and dissection of the breast vein during abdominal surgery

During abdominal surgery, the last two nipples on the right side of the animal were used as anatomical reference to locate the explant position during its surgical removal.

The breast vein is located at the intermammary line. This situation required a careful dissection to avoid accidental damage during implantation surgery. In one case, the vein was damaged and resulted in an extensive bleeding that was resolved with a hemostatic stitch; however, the animal presented postoperatively an abdominal wall hematoma that was resolved spontaneously after few days.

#### Blood extraction from the ear artery

Blood withdrawal from the ear artery may be a difficult procedure. Blood was obtained in 79 occasions. In 6 cases (4.74%) we experienced difficulties that led to the collection of insufficient blood volume to complete the studies. These difficulties occurred in both groups.

Surgical variables, clinical findings during the animal follow-up and macroscopic study of explants are described in Table 1.

**Table 1:** Surgical variables, clinical findings during the animal follow-up and macroscopic study of explants.

	Control (PP) group (N = 10)	Experimental (hADM) group (N = 10)	*p*
Surgical time of implant surgery	75′ (SD* = 37)	80′ (SD* = 35)	0.760
Surgical complications of implant surgery	10%	0	0.305
*Clinical findings during the animal monitoring*			
Abdominal wound infection	30%	10%	0.582
Dirty genitalia	10%	30%	0.582
Stereotypes harm lesions	20%	20%	1.000
Abdominal mesh extrusion	20%	0	0.474
Accidental facial injury	10%	0	1.000
Death (normal autopsy)	10%	0	1.000
Abdominal wound hematoma	10%	0	1.000
Average weight gain	884 g	714.5 g	0.641
Vaginal mesh extrusion	33%	0	0.024
Abdominal mesh extrusion	11%	0	0.474
Chronic infection signs in abdomen location (Fig. 2)	33%	10%	0.303
Chronic infection signs in vaginal location	11%	10%	1.000
Vaginal graft not visible	0	40%	0.014

*SD  Standard deviation.

**Figure 2:**
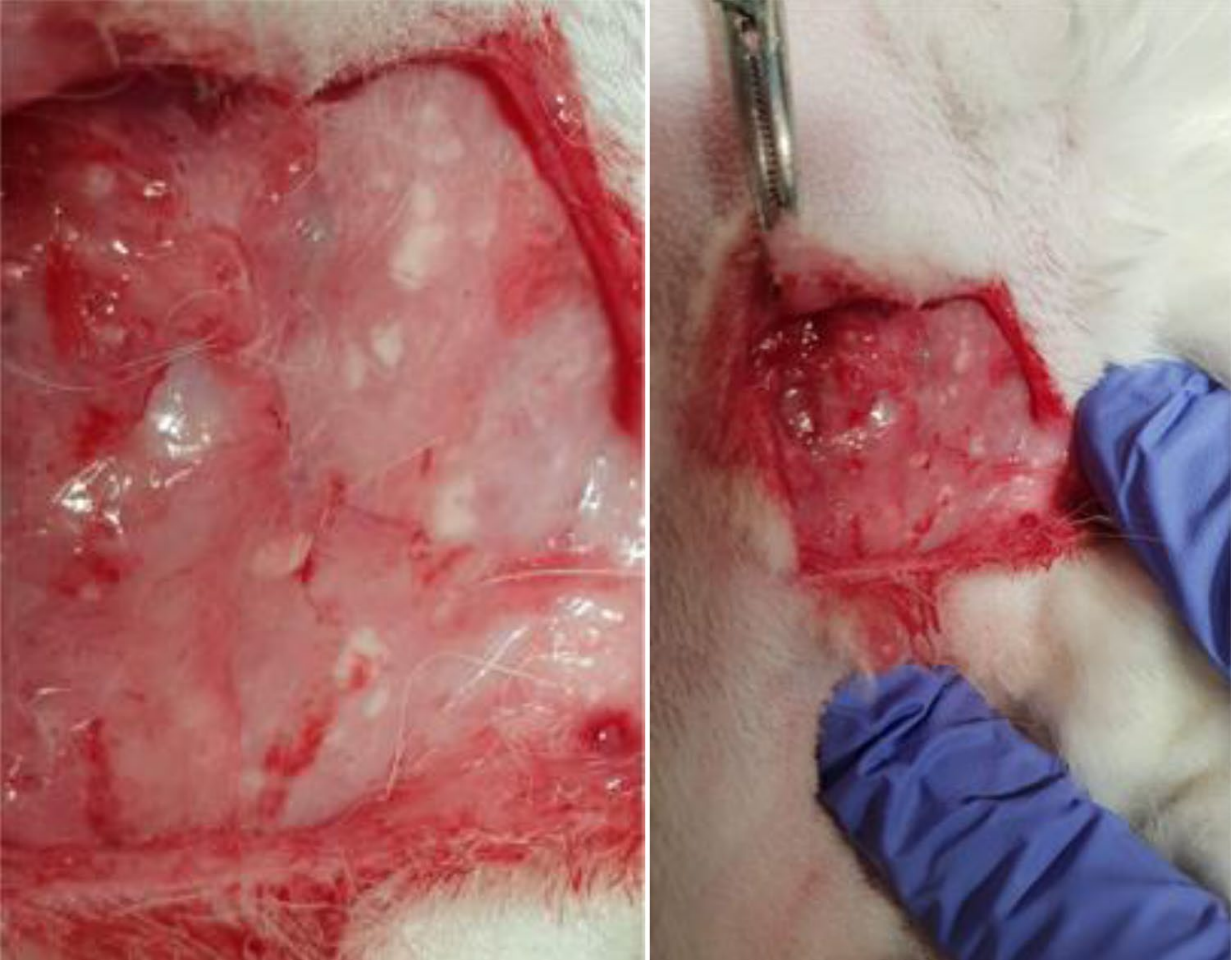
Multiple purulent collections arranged around the mesh, during explant removal surgery.

### Surgical complications of implant surgery

Only one animal suffered a mild hemorrhage in the subcutaneous tissue during abdominal surgery, which was resolved with a hemostatic suture.

### Clinical complications during follow-up

Clinical complications occurred, and actions taken during follow-up and are described in Table 2. The Grimace scale was 0 in all animals and in all evaluations during follow-up.

**Table 2:** Incidences at follow-up.

*Control group*	
A. Three cases (30%) of abdominal surgical wound infection. Resolved with antibiotic treatment	
B. One case (10%) of filthiness in the genital area. No intervention needed	
C. Two cases (20%) of minor self-inflicted damage. Resolved with antiseptic and environmental enrichment measures	
D. Two cases (20%) of extrusion of the abdominal mesh. In one there was spontaneous resolution (correct subcutaneous location of the mesh at the time of euthanasia). In the second case the extrusion persisted	
E. One case (10%) of minor facial injuries due to an accidental incorrect position of the protective collar on the first postoperative day. Resolved favourably with antiseptic measures	
F. One case (10%) of death in the control group, 58 days after surgery. The necropsy showed no complications at the mesh level or other pathological findings of interest	
G. One case (10%) of abdominal wound hematoma which was spontaneously resolved	
*Experimental group*	
A. One case (10%) of abdominal wound infection. Resolved spontaneously	
B. Three cases (30%) of filthiness in the genital area. Resolved in one case by shaving and extensive washing under sedation	
C. Two cases (20%) of minor self-inflicted damage. Resolved with antiseptic and environmental enrichment measures	

## Discussion

This research has evaluated the feasibility of the rabbit as an animal model for the study of biological grafts placed in the abdominal and vaginal locations, and it shows that the NZW is a good model for studying the behaviour of biomaterials in either locations.

The hADM used in this study is an acellular biological matrix, obtained from human dermis, produced to improve the biocompatibility of grafts over that of current synthetic alternatives. hADM is free of allergens, DNA and other pathogens. In this study, the hADMs were implanted in the abdominal fascia and in the vaginal submucosa layer of rabbits. The aim was to evaluate clinical complications during and after graft implantation surgery and macroscopic findings after graft explantation surgery, in different in vivo settings. We used the rabbit as a model on the basis of previous publications, as well as on the characteristics of the animal: adequate life expectancy for the duration of the study; perineal musculature associated with the urogenital tract like humans; enough vaginal ability to perform a vaginal and abdominal implant at the same time; easy and economical animal accommodation; and the availability of investigators trained in handling these animal species.

Other animal models besides the rabbit have been used to study biomaterials in urogynecology^17^. Rats^32–40^ were used; however, due to its small body size studies at vaginal level are very difficult to be performed. The same for mice^18^, where most studied materials are implanted subcutaneously, rather than vaginally^41–43^.

Reviewing studies with large animal models, authors such as Endo et al.^44^, Tayrac et al.^45^, or Feola et al.^46^ studied biological prostheses at vaginal level in sheep. Endo M. compared a cross-linked acellular collagen matrix with a PP mesh, placed simultaneously at vagina and abdomen, also demonstrating greater degradation of vaginal implants (70%). Tayrac compared a noncoated PP mesh against a coated PP mesh with an absorbable hydrophilic film, placed vaginally. In this case, an increase in vaginal exposure rate was also demonstrated in the noncoated PP group (33.3%). Finally, Feola A. compared a PP mesh, a collagen-coated mesh and the native tissue implanted in the vagina and abdomen. This study also showed 22% vaginal erosion rate associated with PP mesh group. These results are consistent with our findings. However, ewes are a more complicated and expensive model that many research centers cannot afford.

Regarding the pig model^47^, the drawbacks are similar to those of sheep: although it has enough size to perform vaginal surgery, and its anatomy is appropriated to the human being, the time required to perform the explants (180 days) makes the sows to increase their weight over 150 kg, which means that the handling of these animals and the costs of the study, even using minipigs, preclude their use in some groups. In the case of the dogs^48^ and NHP, one should add the ethical and legal conflicts concerns.

There are several authors that have used the rabbit model to study different biomaterials in gynecology^21–31^; therefore, we strongly believe that the rabbit is a good model for the study of biomaterials for abdominal and vaginal application.

Graft implantation at the level of the abdominal subcutaneous tissue and in the anterior and posterior vaginal submucosa layer of rabbits was technically simple, and it was associated with very minor surgical complications. However, adequate exposure of the vaginal field is difficult due to its small size. Hence, appropriated training of an assistant and the specific surgical material (suitable for microsurgery) are needed.

Ear blood extraction was also challenging, especially after successive extractions in the same animal because of the narrowing of the vascular lumen secondary to consecutive punctures. Therefore, it is advisable to have the help of trained personnel to perform this technique. Another cause of difficulty in blood withdrawal is the arterial vasoconstriction associated with the decrease in the body temperature of the animals, as well as pain at the puncture site if adequate anesthesia is not achieved.

Complications during the clinical monitoring consisted in minor facial injuries due to accidental incorrect position of the protective collar. To avoid other similar types of injury, protective collars were removed after observing there were no self-inflicted injuries in the surgical wound area. Animals did not show signs of pain during follow-up, so we concluded that a quick, low-invasive, and uncomplicated surgical manipulation is associated with low postoperative pain allowing the avoidance of protective collar placement.

In both groups, stereotypical self-injuries appeared, so it is very important to add environmental enrichment measures in these animals. It is also very important to maintain strict hygiene measures to avoid complications derived from dirtiness.

The occurrence of graft-related complications of implants showed a very different behavior between two groups, especially in the vaginal location.

The clinical complications associated with the graft (wound infection and exposure) were more common in the control (PP synthetic mesh) group, especially in the vaginal location where mesh exposure occurred in 33% of cases (*p* = 0.024). Conversely, in the experimental group, macroscopic hADM degradation at the vaginal level occurred in 40% of cases as compared with 0% in the PP group (*p* = 0.014) whereas in the abdominal location the macroscopic characteristics of the hADM graft remained intact in all cases. These results are consistent with the publications of: Hilger et al.^25^, Pierce et al.^27^ and Higgins et al.^49^. Hilger compared human dermis, porcine dermis, porcine collagen-coated PP mesh, and autologous fascia, implanted in the abdomen and the vagina of a rabbit, also demonstrating greater degradation of the implants in the vaginal location. Pierce compared PP mesh with porcine dermis placed in the vagina and abdomen in a rabbit and observed a 30% degradation rate of the biological graft in the vaginal location. Higgins studied the behavior of PP mesh at the vaginal level in relation to estrogenic levels of a rabbit, demostrating 18% erosion rate in hypoestrogenic group.

Therefore, these latter results show that the rabbit model mimics what is actually observed in humans: graft materials behave differently when implanted in the abdominal wall (i.e., for hernia repair) or in the vaginal submucosa (i.e., for pelvic reconstructive surgery)^50,51^. More extrusion of PP was observed in the vaginal location, whereas more degradation of the hADM was observed in the same location. Higher extrusion suggests a greater inflammatory response after PP implants; while high degradation of hADM suggests better biocompatibility but questions the long-term efficacy for pelvic surgery. The rabbit model allows the study of potential reasons that lead to these differences. Further analysis based on the inflammatory response to different materials in different locations observed in the present study will follow in the future to enable a better understanding of the whole process and to help guiding the development of biomaterials to be used in a human clinical scenario.

The main limitation of the project is the translation of the results from an animal model to a human situation. In this specific case hADM is a heterologous matrix to the rabbit, since is prepared from human material; therefore studies are needed to verify cross-species effects.

None withstanding this is the first experimental model approximation, subsequent clinical studies in women with this hADM will be necessary to verify the results obtained.

## Conclusions

The NZW rabbit is a good model for assessing materials to be used as grafts for pelvic reconstructive surgery and vaginal surgery. The hADM is associated with fewer clinical complications, as well as better macroscopic tissue integration, compared to PP mesh. Additional research is needed to investigate the long-term safety and efficacy of hADM used in women for pelvic reconstructive surgery.
